# Supplementary material for: Type-I Hemins and Free Porphyrins from a Western Australian Sponge Isabela sp
Source: Mar Drugs. 2023 Jan 3;21(1):41. doi: 10.3390/md21010041 (PMC9865862; doi:10.3390/md21010041)
Supplement: Supplementary file 1 [file marinedrugs-21-00041-s001.zip › marinedrugs-2123876-supplementary/Isabela_SI_11122022/Isabellihemin_A.pdf]

SS290419_2

Olex2

Report created with ReportPlus

R_1=11.47%

Crystal Data and Experimental

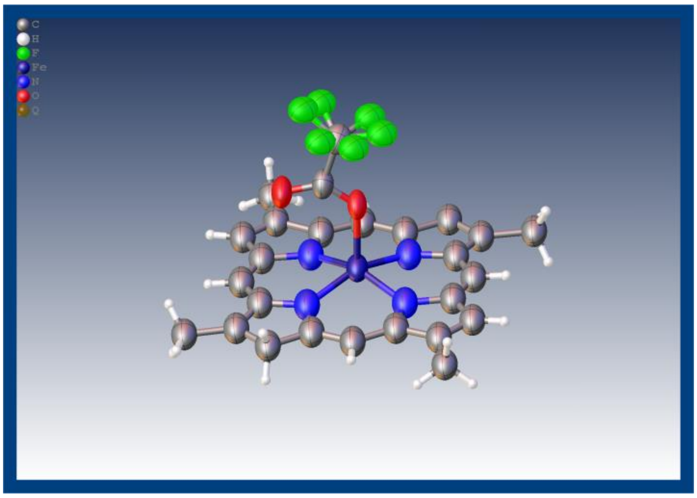

Experimental. Single clear light colourless block-shaped crystals of **SS290419_2** were used as supplied. A suitable crystal with dimensions 0.10 × 0.05 × 0.03 mm³ was selected and mounted on a XtaLAB Synergy, Single source at home/near, HyPix diffractometer. The crystal was kept at a steady T = 120.00(14) K during data collection. The structure was solved with the **ShelXT** 2018/2 (Sheldrick, 2018) solution program using dual methods and by using **Olex2** 1.5 (Dolomanov et al., 2009) as the graphical interface. The model was refined with **XL** (Sheldrick, 2008) using full matrix least squares minimisation on $\boldsymbol{F^2}$.

Crystal Data. $C_{25}H_{20}F_{1.5}FeN_4O_{1.5}$, M_r = 484.80, monoclinic, $P2_1/c$ (No. 14), a = 9.6505(10) Å, b = 15.664(2) Å, c = 17.1896(19) Å, β = 100.170(10)°, $\alpha = \gamma$ = 90°, V = 2557.7(5) Å³, T = 120.00(14) K, Z = 4, Z' = 1, μ(Cu K_α) = 5.029, 13005 reflections measured, 1627 unique (R_{int} = 0.1153) which were used in all calculations. The final wR_2 was 0.3316 (all data) and R_1 was 0.1147 (I≥2 σ(I)).

**Compound**	**SS290419_2**
Formula	$C_{25}H_{20}F_{1.5}FeN_4O_{1.5}$
$D_{calc.}$/ g cm^{-3}	1.259
μ/mm^{-1}	5.029
Formula Weight	484.80
Colour	clear light colourless
Shape	block-shaped
Size/mm³	0.10×0.05×0.03
T/K	120.00(14)
Crystal System	monoclinic
Space Group	$P2_1/c$
a/Å	9.6505(10)
b/Å	15.664(2)
c/Å	17.1896(19)
α/°	90
β/°	100.170(10)
γ/°	90
V/Å³	2557.7(5)
Z	4
Z'	1
Wavelength/Å	1.54184
Radiation type	Cu K_α
Θ_{min}/°	3.846
Θ_{max}/°	41.048
Measured Refl's.	13005
Indep't Refl's	1627
Refl's I≥2 σ(I)	1237
R_{int}	0.1153
Parameters	165
Restraints	119
Largest Peak	0.676
Deepest Hole	-0.523
GooF	1.125
wR_2 (all data)	0.3316
wR_2	0.3090
R_1 (all data)	0.1416
R_1	0.1147

Structure Quality Indicators

**Reflections:**	d min (Cu\a) 2Θ=82.1° 1.17	I/σ(I) 13.3	Rint 11.53%	Full 82.1° 98.1
**Refinement:**	Shift 0.001	Max Peak 0.7	Min Peak -0.5	GooF 1.125

A clear light colourless block-shaped-shaped crystal with dimensions 0.10 × 0.05 × 0.03 mm^3 was mounted. Data were collected using a XtaLAB Synergy, Single source at home/near, HyPix diffractometer operating at T = 120.00(14) K.

Data were measured using ω scans with Cu K_α radiation. The diffraction pattern was indexed and the total number of runs and images was based on the strategy calculation from the program CrysAlisPro 1.171.41.103a (Rigaku OD, 2021). The maximum resolution that was achieved was Θ = 41.048° (1.17 Å).

The unit cell was refined using CrysAlisPro 1.171.41.103a (Rigaku OD, 2021) on 2221 reflections, 17% of the observed reflections.

Data reduction, scaling and absorption corrections were performed using CrysAlisPro 1.171.41.103a (Rigaku OD, 2021). The final completeness is 98.10 % out to 41.048° in Θ. A multi-scan absorption correction was performed using CrysAlisPro 1.171.41.103a (Rigaku Oxford Diffraction, 2021) using spherical harmonics, implemented in SCALE3 ABSPACK scaling algorithm. The absorption coefficient μ of this material is 5.029 mm^{-1} at this wavelength (λ = 1.54184Å) and the minimum and maximum transmissions are 0.612 and 1.000.

The structure was solved and the space group $P2_1/c$ (# 14) determined by the ShelXT 2018/2 (Sheldrick, 2018) structure solution program using using dual methods and refined by full matrix least squares minimisation on $\boldsymbol{F}^2$ using version 2018/3 of **XL** (Sheldrick, 2008). All non-hydrogen atoms were refined anisotropically. Hydrogen atom positions were calculated geometrically and refined using the riding model.

_*smtbx_masks_special_details*: A solvent mask was calculated and 114 electrons were found in a volume of 364$Å^3$ in 1 void per unit cell. This is consistent with the presence of 3[OH2] per Asymmetric Unit which account for 120 electrons per unit cell.

There is a single molecule in the asymmetric unit, which is represented by the reported sum formula. In other words: Z is 4 and Z' is 1.

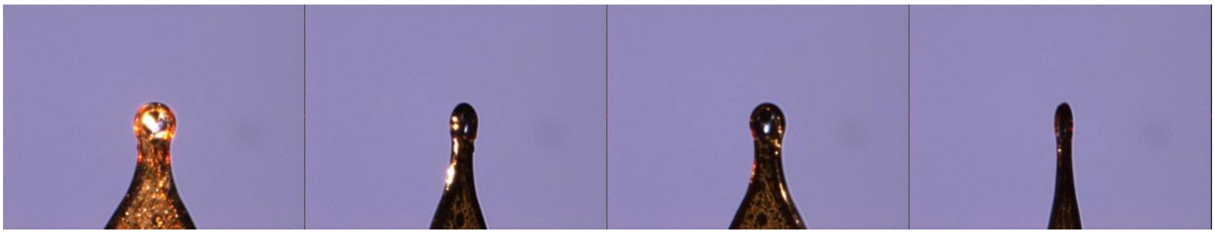

Citations

CrysAlisPro (Rigaku, V1.171.41.103a, 2021)

CrysAlisPro (ROD), Rigaku Oxford Diffraction, Poland (?).

O.V. Dolomanov and L.J. Bourhis and R.J. Gildea and J.A.K. Howard and H. Puschmann, Olex2: A complete structure solution, refinement and analysis program, *J. Appl. Cryst.*, (2009), **42**, 339-341.

Sheldrick, G.M., Crystal structure refinement with ShelXL, *Acta Cryst.*, (2015), **C71**, 3-8.

Sheldrick, G.M., ShelXT-Integrated space-group and crystal-structure determination, *Acta Cryst.*, (2015), **A71**, 3-8.
