# Supplementary material for: Type-I Hemins and Free Porphyrins from a Western Australian Sponge Isabela sp
Source: Mar Drugs. 2023 Jan 3;21(1):41. doi: 10.3390/md21010041 (PMC9865862; doi:10.3390/md21010041)
Supplement: Supplementary file 1 [file marinedrugs-21-00041-s001.zip › marinedrugs-2123876-supplementary/Isabela_SI_11122022/isabellin_A.pdf]

MOGG06

Report created with ReportPlus

R_1=6.21%

Crystal Data and Experimental

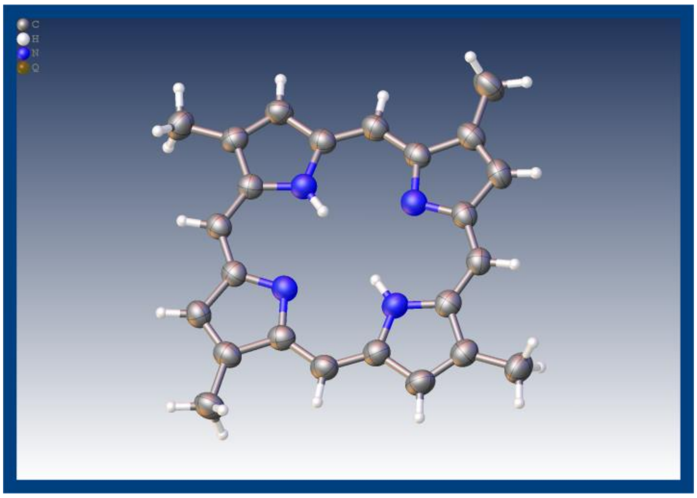

Experimental. Single clear dark red block-shaped crystals of **MOGG06** were used as supplied. A suitable crystal with dimensions 0.08 × 0.05 × 0.04 mm^3 was selected and mounted on a XtaLAB Synergy, Single source at home/near, HyPix diffractometer. The crystal was kept at a steady T = 150.00(10) K during data collection. The structure was solved with the **ShelXT** 2018/2 (Sheldrick, 2018) solution program using dual methods and by using **Olex2** 1.5 (Dolomanov et al., 2009) as the graphical interface. The model was refined with **XL** (Sheldrick, 2008) using full matrix least squares minimisation on ***F*²**.

Crystal Data. $C_{24}H_{22}N_4$, M_r = 366.45, monoclinic, $P2_1/c$ (No. 14), a = 11.7999(5) Å, b = 10.7615(5) Å, c = 14.9068(6) Å, β = 96.745(4)°, α = γ = 90°, V = 1879.83(14) Å^3, T = 150.00(10) K, Z = 4, Z' = 1, μ(Cu K_α) = 0.609, 18158 reflections measured, 3906 unique (R_{int} = 0.0539) which were used in all calculations. The final wR_2 was 0.2031 (all data) and R_1 was 0.0621 (I≥2 σ(I)).

**Compound**	**MOGG06**
Formula	$C_{24}H_{22}N_4$
$D_{calc.}$/ g cm^{-3}	1.295
μ/mm^{-1}	0.609
Formula Weight	366.45
Colour	clear dark red
Shape	block-shaped
Size/mm^3	0.08×0.05×0.04
T/K	150.00(10)
Crystal System	monoclinic
Space Group	$P2_1/c$
a/Å	11.7999(5)
b/Å	10.7615(5)
c/Å	14.9068(6)
α/°	90
β/°	96.745(4)
γ/°	90
V/Å^3	1879.83(14)
Z	4
Z'	1
Wavelength/Å	1.54184
Radiation type	Cu K_α
Θ_{min}/°	3.772
Θ_{max}/°	78.111
Measured Refl's.	18158
Indep't Refl's	3906
Refl's I≥2 σ(I)	2515
R_{int}	0.0539
Parameters	257
Restraints	0
Largest Peak	0.960
Deepest Hole	-0.195
GooF	1.219
wR_2 (all data)	0.2031
wR_2	0.1805
R_1 (all data)	0.0968
R_1	0.0621

Structure Quality Indicators

**Reflections:**	d min (Cu\a) 2Θ=156.2° 0.79	I/σ(I) 19.3	Rint 5.39%	Full 135.4° 97% to 156.2° 99.7
**Refinement:**	Shift 0.000	Max Peak 1.0	Min Peak -0.2	GooF 1.219

A clear dark red block-shaped-shaped crystal with dimensions 0.08 × 0.05 × 0.04 mm^3 was mounted. Data were collected using a XtaLAB Synergy, Single source at home/near, HyPix diffractometer operating at T = 150.00(10) K.

Data were measured using ω scans with Cu K$_\alpha$ radiation. The diffraction pattern was indexed and the total number of runs and images was based on the strategy calculation from the program CrysAlisPro 1.171.41.103a (Rigaku OD, 2021). The maximum resolution that was achieved was Θ = 78.111° (0.79 Å).

The unit cell was refined using CrysAlisPro 1.171.41.103a (Rigaku OD, 2021) on 5972 reflections, 33% of the observed reflections.

Data reduction, scaling and absorption corrections were performed using CrysAlisPro 1.171.41.103a (Rigaku OD, 2021). The final completeness is 99.70 % out to 78.111° in Θ. A multi-scan absorption correction was performed using CrysAlisPro 1.171.41.103a (Rigaku Oxford Diffraction, 2021) using spherical harmonics, implemented in SCALE3 ABSPACK scaling algorithm. The absorption coefficient μ of this material is 0.609 mm^{-1} at this wavelength (λ = 1.54184Å) and the minimum and maximum transmissions are 0.844 and 1.000.

The structure was solved and the space group $P2_1/c$ (# 14) determined by the ShelXT 2018/2 (Sheldrick, 2018) structure solution program using using dual methods and refined by full matrix least squares minimisation on $\boldsymbol{F^2}$ using version 2018/3 of **XL** (Sheldrick, 2008). All non-hydrogen atoms were refined anisotropically. Hydrogen atom positions were calculated geometrically and refined using the riding model.

There is a single molecule in the asymmetric unit, which is represented by the reported sum formula. In other words: Z is 4 and Z' is 1.

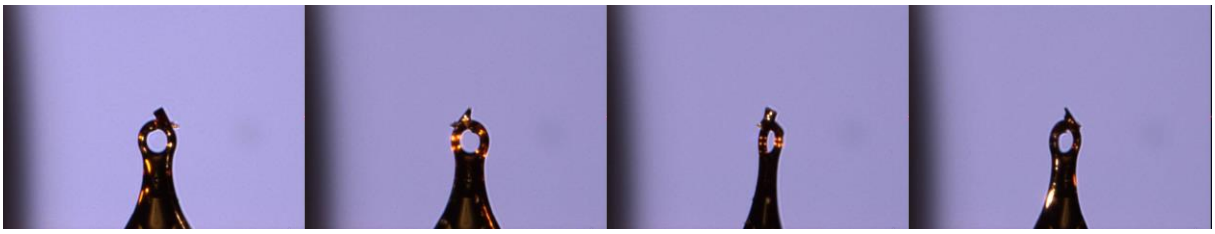

Citations

CrysAlisPro (Rigaku, V1.171.41.103a, 2021)

CrysAlisPro (ROD), Rigaku Oxford Diffraction, Poland (?).

O.V. Dolomanov and L.J. Bourhis and R.J. Gildea and J.A.K. Howard and H. Puschmann, Olex2: A complete structure solution, refinement and analysis program, *J. Appl. Cryst.*, (2009), **42**, 339-341.

Sheldrick, G.M., Crystal structure refinement with ShelXL, *Acta Cryst.*, (2015), **C71**, 3-8.

Sheldrick, G.M., ShelXT-Integrated space-group and crystal-structure determination, *Acta Cryst.*, (2015), **A71**, 3-8.
