# Supplementary material for: Type-I Hemins and Free Porphyrins from a Western Australian Sponge Isabela sp
Source: Mar Drugs. 2023 Jan 3;21(1):41. doi: 10.3390/md21010041 (PMC9865862; doi:10.3390/md21010041)

Type-I Hemins and Free Porphyrins from a Western Australian Sponge *Isabela* sp.

Supporting Information

Samuele Sala[*†⊥], Stephen A. Moggach[†], Gareth L. Nealon[†], Jane Fromont[‡], Oliver Gomez[‡], Daniel Vuong[§], Ernest Lacey[§] and Gavin. R. Flematti[*†].

†School of Molecular Sciences, The University of Western Australia, Crawley, WA 6009, Australia

⊥Australian National Phenome Centre and Centre for Computational and Systems Medicine, Health Futures Institute, Murdoch University, Harry Perkins Building, Perth, WA 6150, Australia

‡Collection and Research, Western Australian Museum, Welshpool, WA 6106, Australia

§Microbial Screening Technologies Pty. Ltd., Smithfield, NSW 2164, Australia

*To whom correspondence should be addressed

SS: Current address: Australian National Phenome Centre and Centre for Computational and Systems Medicine, Health Futures Institute, Murdoch University, Harry Perkins Building, Perth, WA 6150, Australia

T: +61 4 0116 9864; E: Samuele.Sala@murdoch.edu.au

GRF: The University of Western Australia; Perth, WA, 6009, Australia; T: +61 8 64884461; E: gavin.flematti@uwa.edu.au;

Contents

S1. ^{1}H NMR of isabellin A (**1a**) (600 MHz, $CDCl_3$) 3
S2. COSY NMR of isabellin A (**1a**) (600 MHz, $CDCl_3$) 4
S3. HSQC NMR of isabellin A (**1a**) (600 MHz, 150 MHz, $CDCl_3$) 5
S4. HMBC NMR of isabellin A (**1a**) (600 MHz, 150 MHz, $CDCl_3$) 6
S5. ^{1}H NMR of isabellin B (**2a**) (600 MHz, $(CH_3)_2CO$) 7
S6. NOESY NMR of isabellin B (**2a**) (600 MHz, $(CH_3)_2CO$) 8
S7. NOESY NMR of isabellin B (**2a**) (600 MHz, $(CH_3)_2CO$), expansion 9

S1. ^{1}H NMR of isabellin A (**6.1a**) (600 MHz, $CDCl_3$)

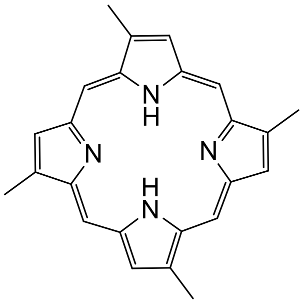

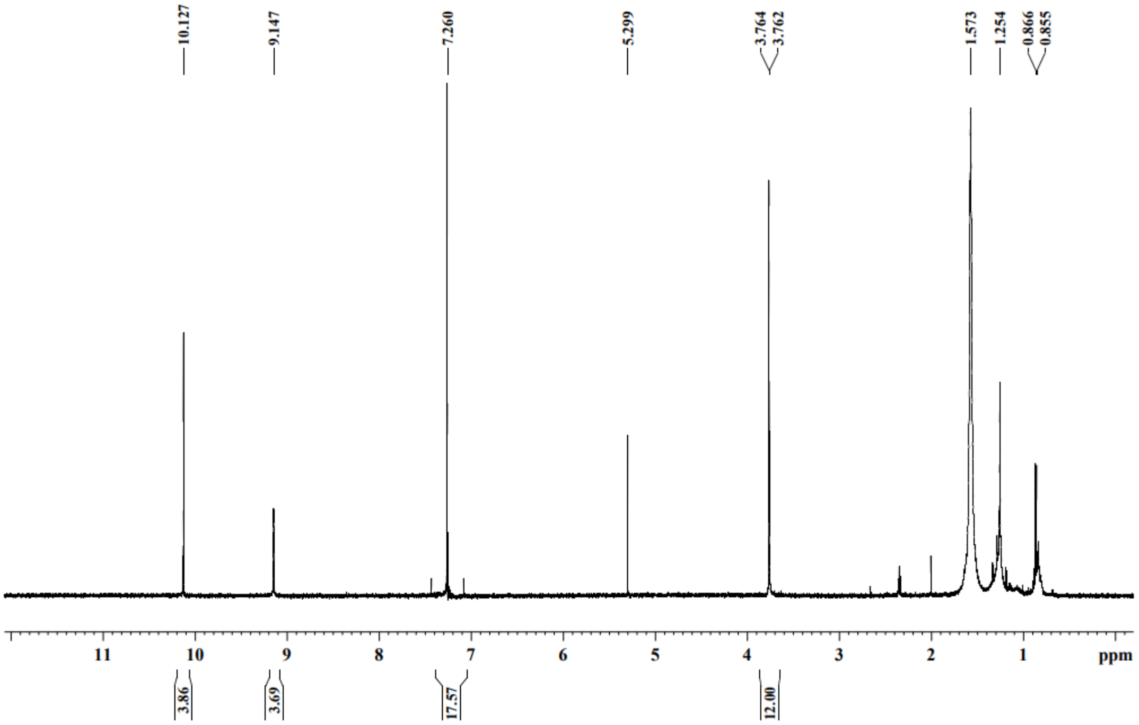

S2. COSY NMR of isabellin A (**6.1a**) (600 MHz, $CDCl_3$)

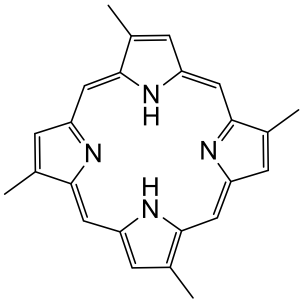

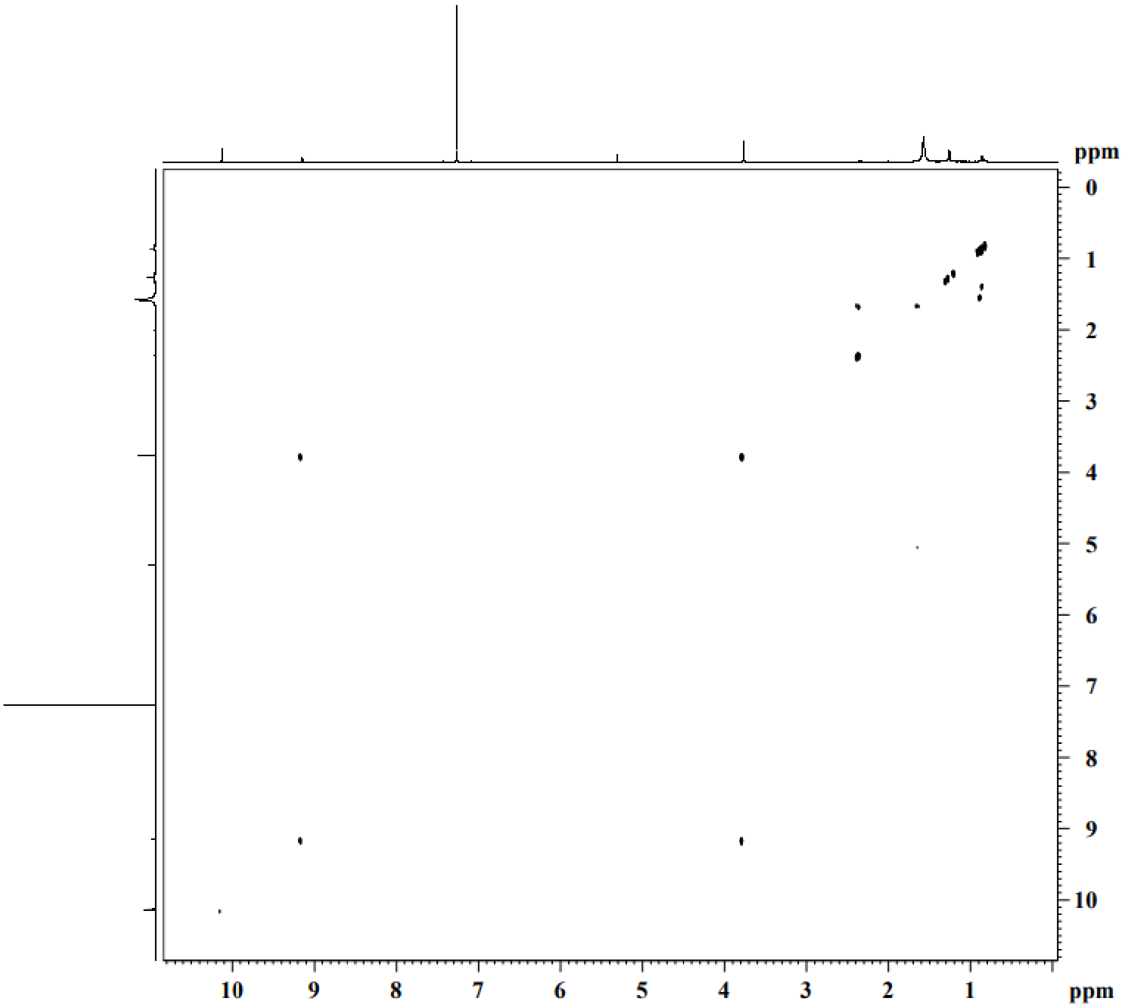

S3. HSQC NMR of isabellin A (**6.1a**) (600 MHz, 150 MHz, $CDCl_3$)

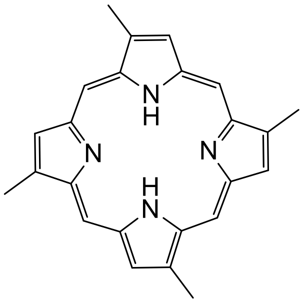

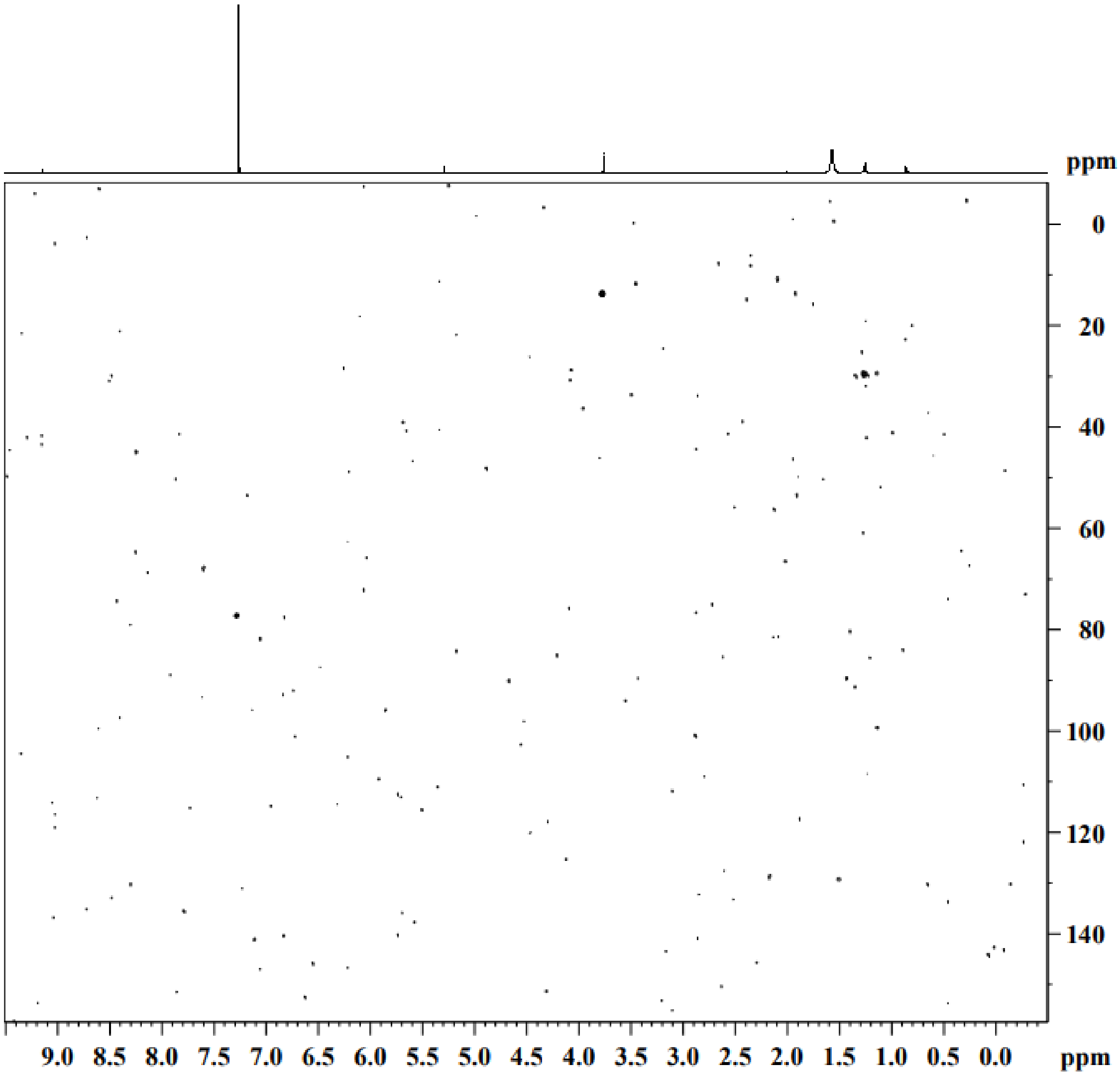

S4. HMBC NMR of isabellin A (**6.1a**) (600 MHz, 150 MHz, $CDCl_3$)

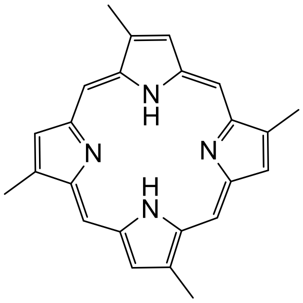

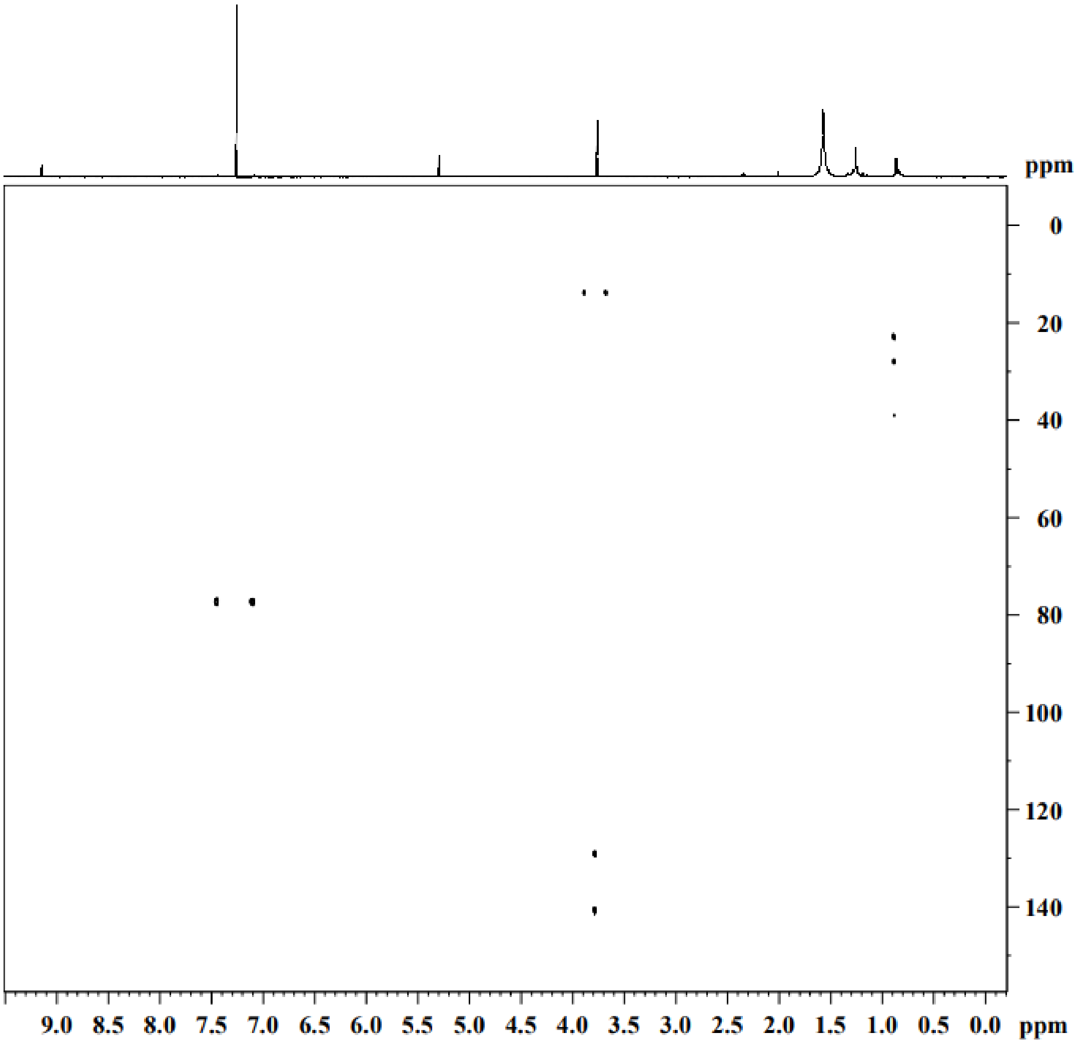

S5. ^{1}H NMR of isabellin B (**6.2a**) (600 MHz, $(CH_3)_2CO$)

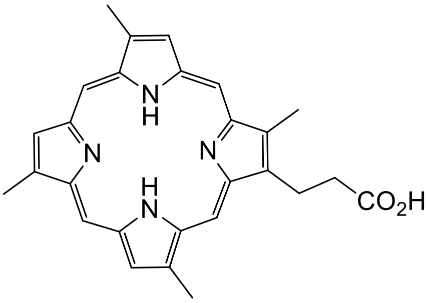

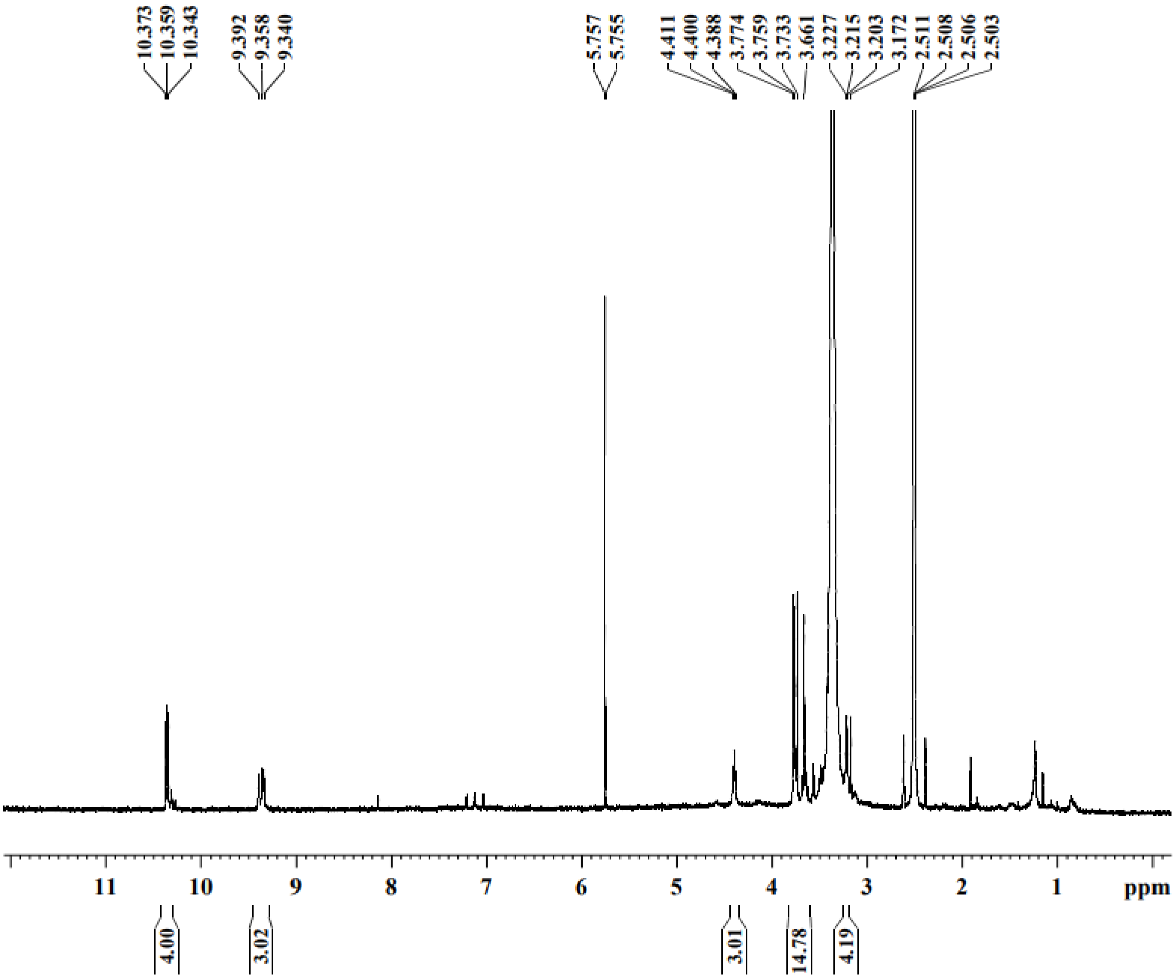

S6. NOESY NMR of isabellin B (**6.2a**) (600 MHz, $(CH_3)_2CO$)

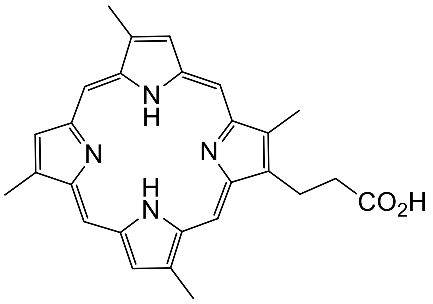

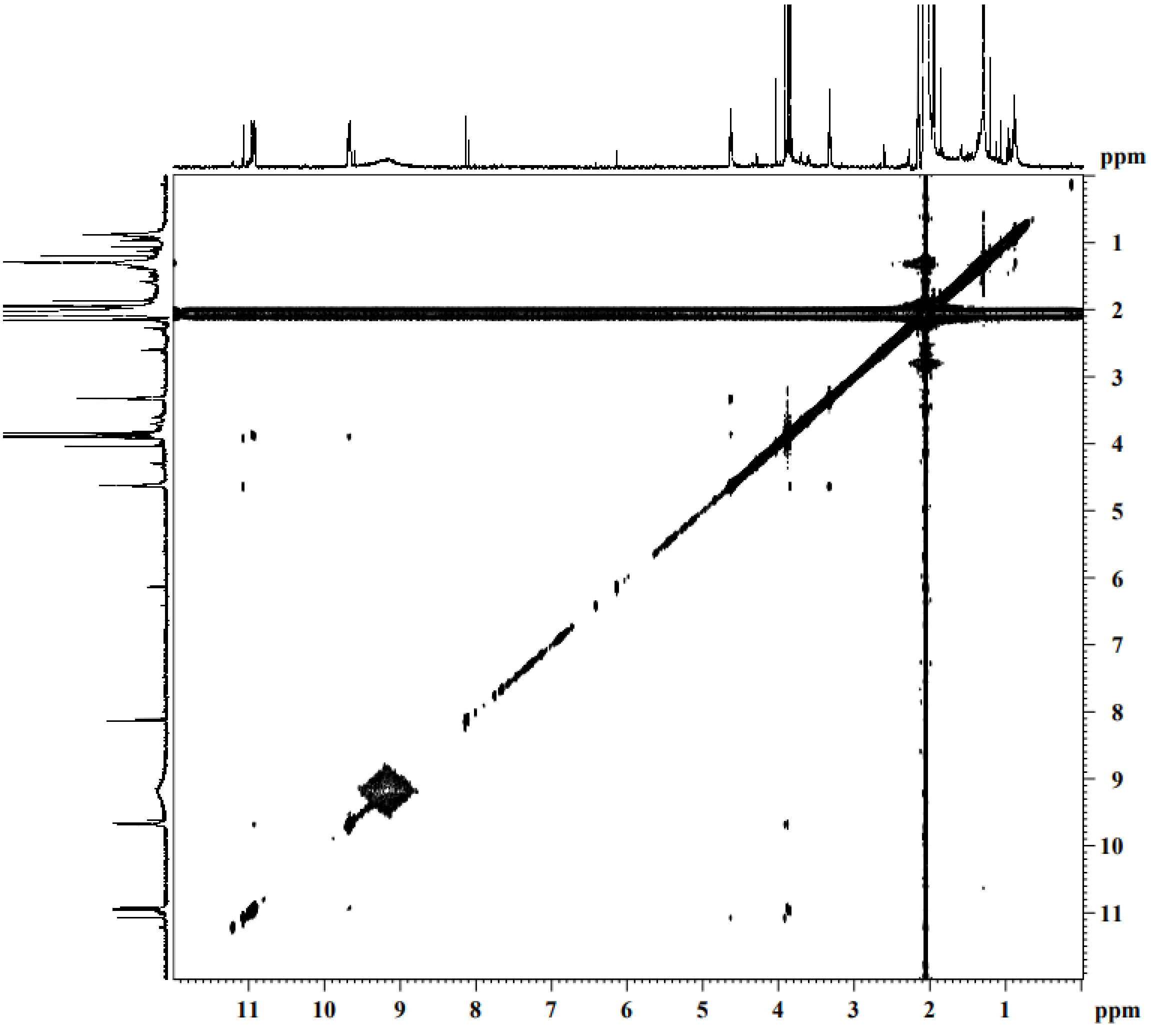

S7. NOESY NMR of isabellin B (**6.2a**) (600 MHz, $(CH_3)_2CO$), expansion

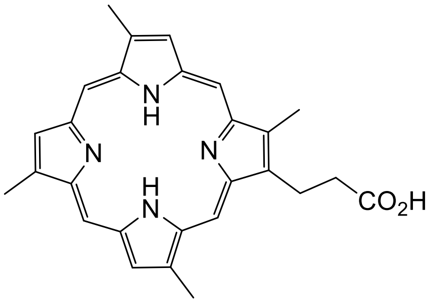

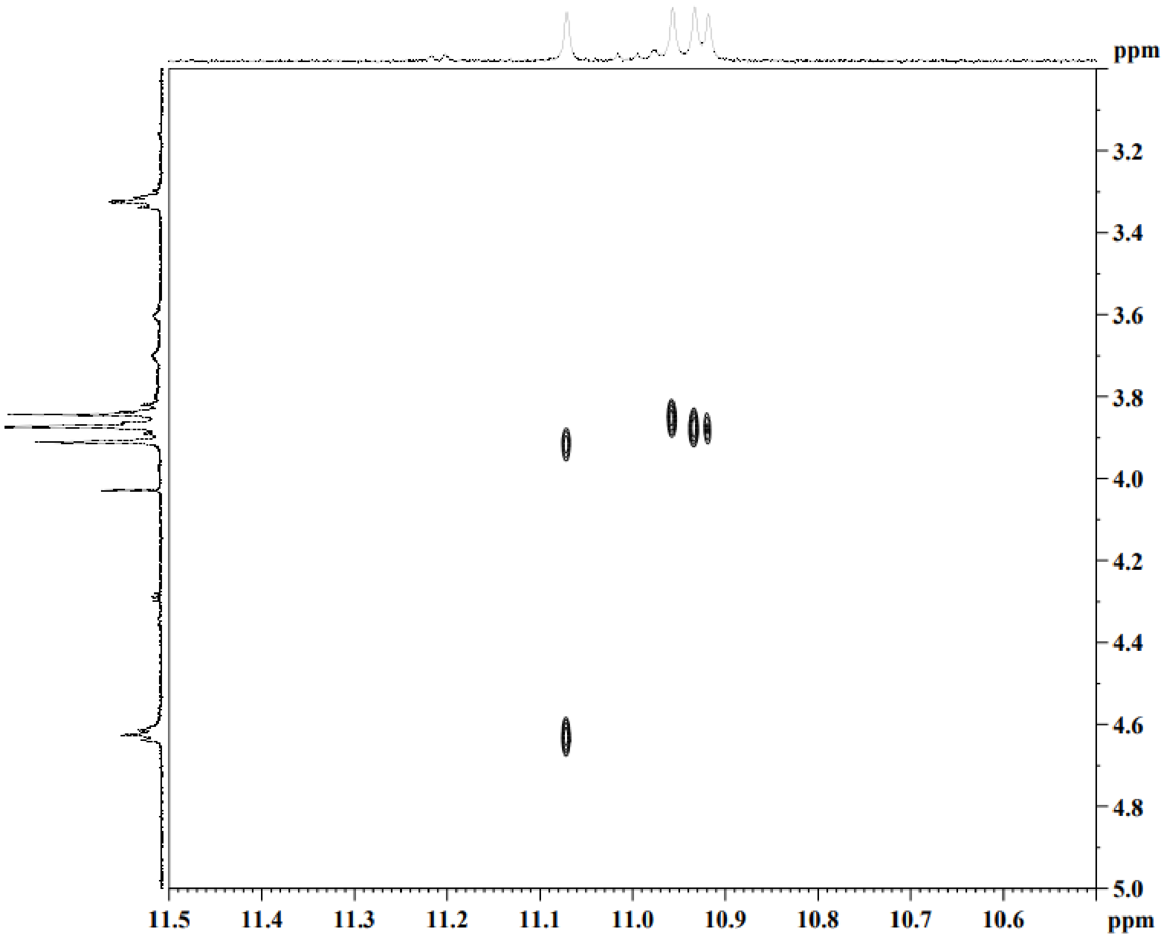

Supplement: Supplementary file 1 [file marinedrugs-21-00041-s001.zip › marinedrugs-2123876-supplementary/Isabela_SI_11122022/Supporting_Information_Isabela_11122022.pdf]
